# Radiofrequency ablation of renal tumour in the supine position using a dorsal-space mat

**DOI:** 10.1093/bjrcr/uaaf025

**Published:** 2025-04-11

**Authors:** Yuya Koike, Kenji Motohashi, Naoki Kuwabara, Takahiro Miyauchi, Masahiro Okada

**Affiliations:** Department of Radiology, Nihon University School of Medicine, Tokyo 173-8610, Japan; Department of Radiology, Nihon University School of Medicine, Tokyo 173-8610, Japan; Department of Radiology, Nihon University School of Medicine, Tokyo 173-8610, Japan; Department of Radiology, Nihon University School of Medicine, Tokyo 173-8610, Japan; Department of Radiology, Nihon University School of Medicine, Tokyo 173-8610, Japan

**Keywords:** radiofrequency ablation, supine position, procedural space, dorsal approach

## Abstract

Radiofrequency ablation (RFA) of renal tumours is one of the major procedures in nonvascular interventional radiology, but it is difficult to perform in patients who are limited to the supine position. In this technical note, we present 2 cases in which RFA of renal tumours was performed via a dorsal approach in patients in the supine position by using a mat with a space for the procedure. Three polyorefin mats, 15 cm high and of different lengths, were arranged on the CT table to provide space for the procedure. Each patient was placed supine on these mats, and RFA of the renal tumour was performed via a dorsal approach under CT fluoroscopy. Two patients successfully underwent RFA via the dorsal approach with no postural distress during the 40- and 75-min procedures. There were no complications related to the procedure. The dorsal approach using a mat with space for the procedure is particularly useful for performing renal tumour RFA in patients with limited supine positioning. The method of creating a space on the patient’s dorsal side by arranging 3 polyorefin mats is reproducible in terms of simplifying the approach and is expected to be applied to other nonvascular interventions.

## Introduction

Radiofrequency ablation (RFA) for renal tumours can be a particularly effective treatment for patients who are unable or unwilling to tolerate conventional surgical approaches or active surveillance.^1^ The procedure typically takes 90^2^ to 120 min^3^ and requires a dorsal puncture approach, which requires adjustment of the patient’s position, such as prone or lateral position.^4^ However, patients who have difficulty maintaining the prone position are not indicated for this procedure, or it may result in interruption of the procedure. Therefore, we proposed a method to enable a dorsal puncture approach while the patient is kept in the supine position. While wedges and pillows can be used to provide a puncture route, we present a simpler and more reproducible method that uses a mat with a space for the procedure.

## Technique

According to a preliminary study evaluating images from a previous CT-guided procedure (unpublished data), the body size in axial section images at the level of the target lesion was 328 ± 32 mm wide, 218 ± 23 mm high, and the puncture depth to the target lesion was 64 ± 23 mm. Patients on a 20-cm-high mat were expected to easily collide with the 70-cm-diameter CT gantry, especially with their upper extremities. Based on the above, the height of the mat for the dorsal approach in a supine patient was set at 15 cm.

**Figure 1.**
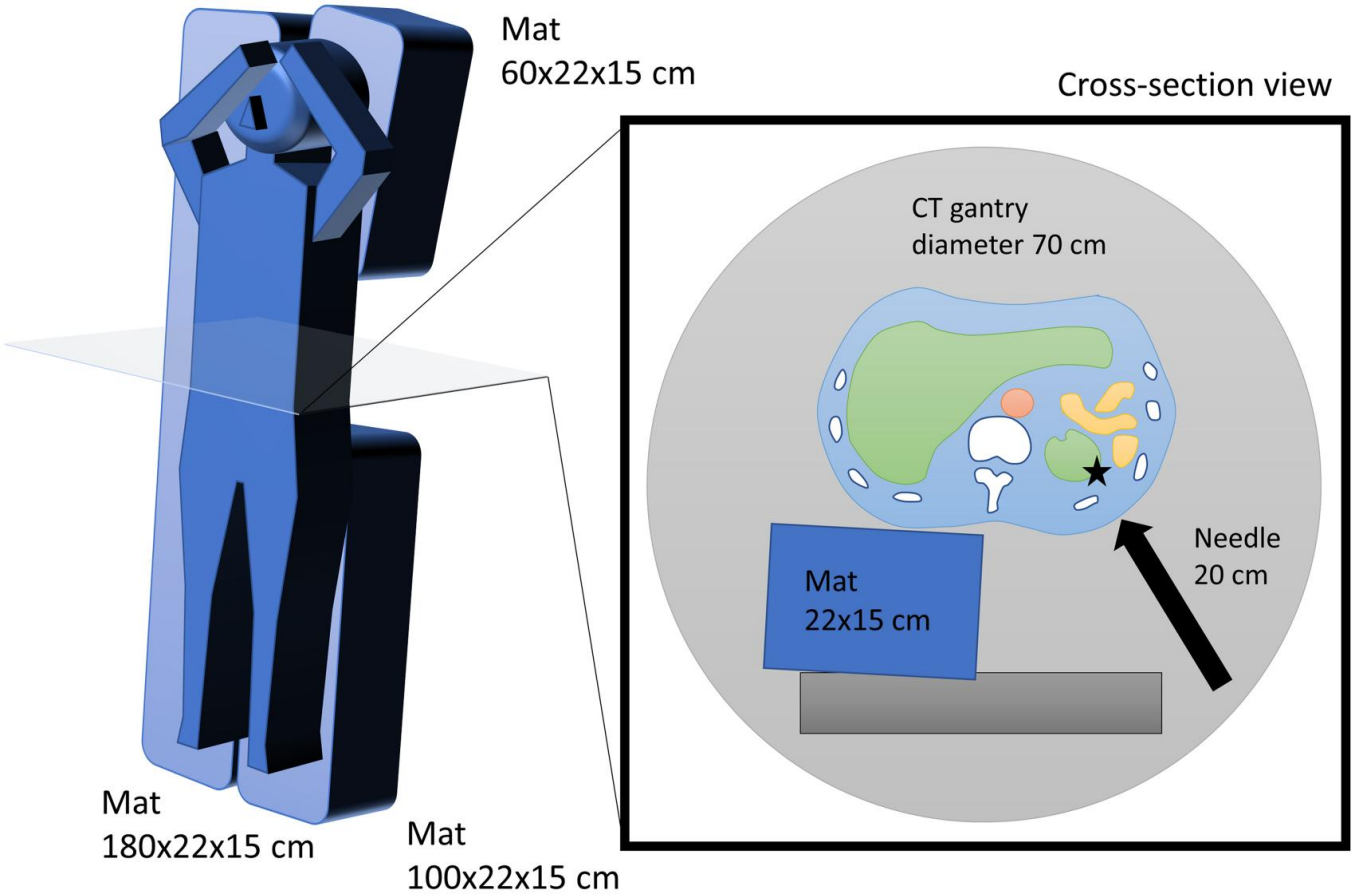
Schematic of a dorsal puncture with the use of a mat with a space for the procedure for a patient confined to the supine position. (Left) Three polyorefin mats are placed on the CT table to create a space on the patient’s dorsal side for the procedure. (Right) In the cross-sectional view, it is possible to approach the lesion (★) from the left dorsal side of a supine patient through the procedural space.

To create space for the procedure on the dorsal side of the supine patient, 3 mats were placed on the CT table. The mats were made of polyorefin and were 180 cm long × 22 cm wide × 15 cm high, 60 cm long × 22 cm wide × 15 cm high, and 90 cm long × 22 cm wide × 15 cm high. Since there is no commercial brand of such mats, they were created by ordering moulded polyorefin for approximately £310/$400 (Marusuzu, Tokyo, Japan), which can be easily reproduced. In addition, the mat is reusable because sheets and drapes are placed on the mat before placing the patient. On the normal side, a long mat was placed from the head to the feet, and on the diseased side, mats were placed from the head to the chest and from the waist to the feet, respectively. This provided a stable supine posture and at the same time created a space for the procedure on the dorsal side of the lumbar region on the diseased side. From this space, it was possible to puncture the lesion from the dorsal side of the patient in the supine position (Figure 1).

## Case 1

**Figure 2.**
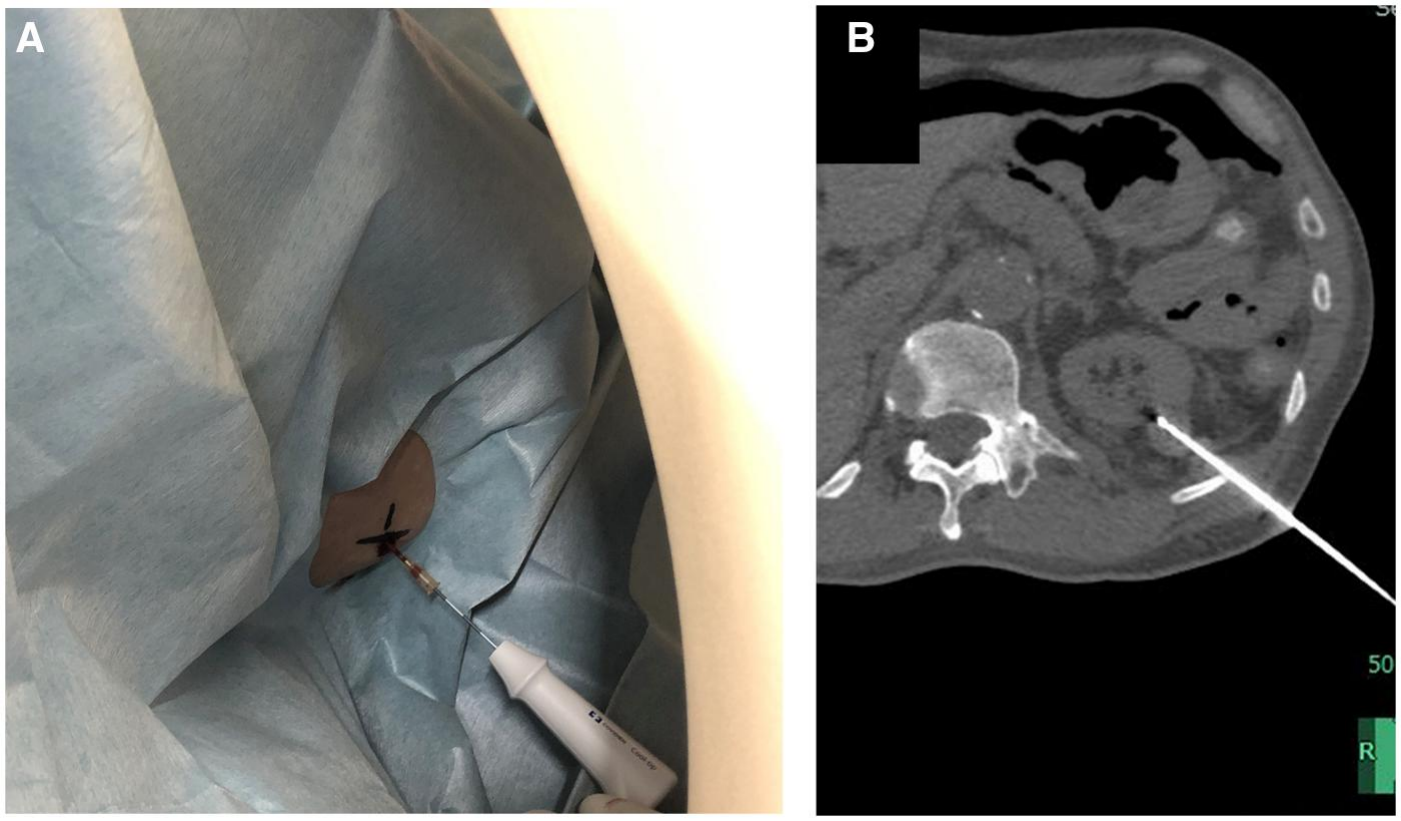
A 61-year-old man undergoing RFA for a right renal tumour. (A) The patient is supine and the RF needle is introduced through the left lateral dorsal aspect. The RF needle does not interfere with the CT gantry or the table. (B) CT image after insertion of the RF needle into the renal tumour using CT fluoroscopic guidance. The polyorefin mat is not visible in normal window setting, but there is space on the patient’s dorsal side.

A 61-year-old male was referred for RFA for a 20 mm diameter left renal tumour. The patient had a history of peritoneal dialysis for about 10 years and had chronic recurrent peritonitis and peritoneal dialysis tube infection for several months. On admission, there were no signs of infection and the C-reactive protein level was below 1, but the patient was difficult to position supine due to tenderness along the peritoneal dialysis tubing. Therefore, RFA was performed via a dorsal puncture approach using a mat with a space for the procedure (Figure 2).

## Case 2

**Figure 3.**
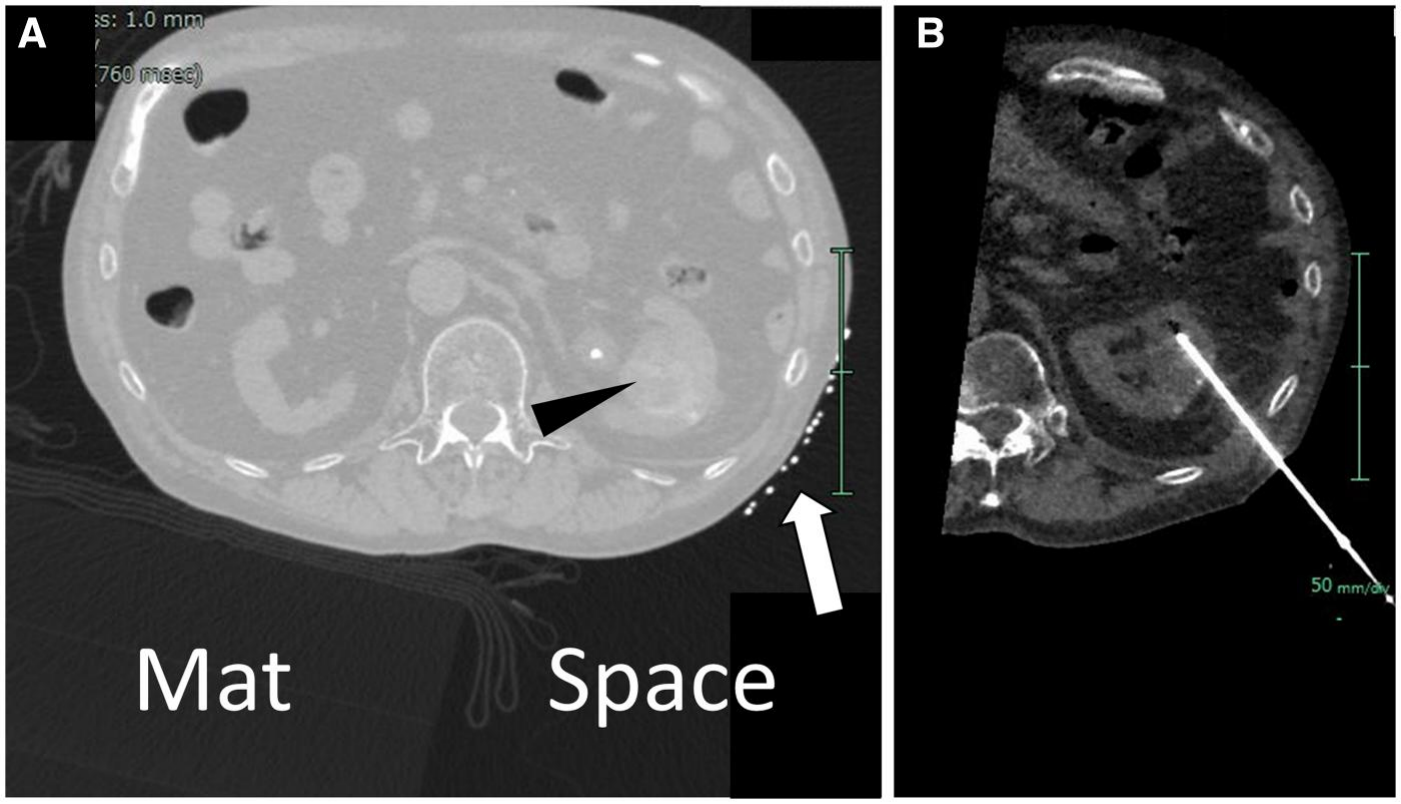
A 69-year-old man undergoing RFA for a left renal tumour. (A) CT image of puncture planning (pulmonary window setting). The polyorefin mat is thinly visible in the pulmonary window setting, revealing the presence of a procedural space. The patient is supine, and a marker (arrow) is placed on the left lateral back. Arrowhead: left renal tumour. (B) CT image during CT fluoroscopic guidance. The RF needle is inserted into the left renal tumour through the procedural space.

A 69-year-old man with severe emphysema and atrial fibrillation was referred for RFA for a 33 mm diameter right renal tumour. Because the renal tumour was close to the renal pelvis, a ureteral catheter was inserted and perfusion was applied. Ablation at 4 sites was planned to obtain sufficient margin, and a procedure time of about 1 h was estimated. The patient had difficulty maintaining the prone position due to shoulder osteoarthritis, and a dorsal puncture approach using a mat with a space for the procedure was chosen (Figure 3).

## Procedure

The RFA procedure was performed percutaneously under conscious sedation and CT fluoroscopy (Brilliance iCT, Philips, Cleveland, OH, United States). The patient was supine on the mat with space for the procedure, and a CT scan was obtained for puncture planning. The scanning parameters were tube voltage of 120 kV, tube current of 40 mA, rotation time of 0.5 s, and slice thickness of 3 × 3.33 mm, with no automatic exposure control. Via a dorsal approach, following local anaesthesia, a single internally cooled electrode (Cool-tip^®^; Covidien, Mansfield, MA, United States) with a 3-cm uninsulated tip was introduced into the renal tumour. To obtain sufficient margins, overlapping ablations were performed at 2 sites in case 1 and at 4 sites in case 2, respectively. Radiofrequency energy was applied for 15 min in case 1 and 43 min in case 2 using an impedance control algorithm during internal electrode cooling. Total procedure time was approximately 40 min for case 1 and 75 min for case 2. During the procedure, each patient’s posture remained stable and there was no deformation or displacement of the 3 mats. Both patients successfully completed the dorsal puncture approach with the use of a mat with a space for the procedure, with no positioning difficulties and no complications.

## Discussion

By using a mat with a designated space for the procedure, we successfully performed RFA for renal tumours while the patient remained in the supine position. Generally, physicians use wedges or pillows to adjust the patient’s position and adjust the puncture route, but it depends on the experience of the physician and the postural stability is not sufficient. The dorsal puncture method, using a mat with a procedure space, is simple, reproducible, and provides postural stability.

In the present method, the basic procedural steps remain the same even when the patient is switched from the prone to supine position by using a mat with a space for the procedure. Since the patient’s head, shoulders, buttocks, and lower extremities are stable on the mat, there is no postural instability due to the space for the procedure. The mats did not deform under the patient’s weight. Therefore, the dorsal puncture approach using a mat with a space for the procedure can be performed safely, and no complications were observed. An additional advantage is that the supine position reduces inflation of the lower lobes of the lungs, which may reduce the risk of lung injury and pneumothorax during puncture of dorsal organs such as the kidneys. A few studies have reported on interventions that devise a puncture approach in patients who are limited to the supine position.^5^^,^^6^ Application of these approaches provides patients who are limited to the supine position with opportunities for treatment and relief from postural distress during the procedure. In patients on ventilators, this may reduce the burden on many staff members to adjust positions. All of the previously reported approaches in the axial direction are useful in selected cases but require physician skill.^5^^,^^6^ On the other hand, the present method is highly reproducible because any physician can perform the dorsal approach by arranging the 3 mats. Furthermore, by adjusting the position of the mats, the method can be applied to any target lesion in any location. It is expected that the dorsal approach using a mat with a space for the procedure will be applicable to other non-vascular procedures, including ultrasound-guided, cone-beam CT-guided, and MRI-guided techniques.

In our preliminary experiments, we investigated mat sizes that could be used in various CT-guided procedures in terms of approach reproducibility and simplicity. A mat height of about 30 cm would be ideal for performing the procedure from the dorsal aspect, but the diameter of the CT gantry and the patient’s body size must be taken into consideration. We often use a 15 cm RF needle (approximately 20 cm in length) to reach renal tumours through the skin, but due to the 70 cm diameter limitation of the CT gantry, we designed a mat with a height of 15 cm. The oblique dorsal approach was feasible, but a dorsal-vertical entry path was not possible.

The following points need to be investigated in future studies. First, the feasibility of the procedure considering the size of the gantry, the patient’s body size, and the length of the needle. Second, the expansion of the indication to procedures other than RFA for renal cancer, such as biopsy and drainage. Third, the evaluation of radiation exposure to the physician, since the physician must support the needle to prevent it from falling out due to its own weight. As for patient exposure, it is not expected to increase considerably even if the overall thickness of the imaging target increases. This is because the tube current time product is fixed without using automatic exposure control, and the polyorefin mat has very high X-ray transparency. Nevertheless, since the imaging target is offset within the gantry, an evaluation of radiation exposure is desirable.

In conclusion, the use of a mat with a space for the procedure allows a simple and reproducible dorsal puncture approach and is a promising method for RFA for renal tumours in patients who are limited to the supine position.

## Data Availability

The data that support the findings of this study are available from the corresponding author, upon reasonable request.
